# Biochemical Characterization of a Novel Endo-1,3-β-Glucanase from the Scallop *Chlamys farreri*

**DOI:** 10.3390/md18090466

**Published:** 2020-09-16

**Authors:** Zhijian Li, Weizhi Liu, Qianqian Lyu

**Affiliations:** 1 MOE Key Laboratory of Marine Genetics and Breeding, College of Marine Life Sciences, Ocean University of China, Qingdao 266003, China; lizhijian11@foxmail.com (Z.L.); liuweizhi@ouc.edu.cn (W.L.); 2 Laboratory for Marine Biology and Biotechnology, Qingdao National Laboratory for Marine Science and Technology, Qingdao 266235, China

**Keywords:** endo-1,3-β-glucanases, marine mollusk, transglycosylation, laminarin

## Abstract

Endo-1,3-β-glucanases derived from marine mollusks have attracted much attention in recent years because of their unique transglycosylation activity. In this study, a novel endo-1,3-β-glucanase from the scallop *Chlamys farreri*, named L_cf_, was biochemically characterized. Unlike in earlier studies on marine mollusk endo-1,3-β-glucanases, L_cf_ was expressed in vitro first. Enzymatic analysis demonstrated that L_cf_ preferred to hydrolyze laminarihexaose than to hydrolyze laminarin. Furthermore, L_cf_ was capable of catalyzing transglycosylation reactions with different kinds of glycosyl acceptors. More interestingly, the transglycosylation specificity of L_cf_ was different from that of other marine mollusk endo-1,3-β-glucanases, although they share a high sequence identity. This study enhanced our understanding of the diverse enzymatic specificities of marine mollusk endo-1,3-β-glucanases, which facilitated development of a unique endo-1,3-β-glucanase tool in the synthesis of novel glycosides.

## 1. Introduction

Endo-1,3-β-glucanases (EC 3.2.1.39), also called laminarinases, can specifically hydrolyze β-1,3-glycosidic bonds in laminarin, whereas they hardly hydrolyze mix-linked β-1,3-1,4-glycosidic bonds in lichenin and cereal β-glucans [1]. The products of hydrolyzing laminarin, glucooligosaccharides, have been demonstrated to have a variety of biological activities, such as anti-diabetic activity [2], stimulating leukocytes to induce the production of cytokines [3], modulating lipid metabolism and intestinal microflora [4], and activating defense responses of plant cells [5,6]. Therefore, suitable endo-1,3-β-glucanases are determinants for the enzymatic preparation of well-defined glucooligosaccharides. In addition to their hydrolytic activity, some endo-1,3-β-glucanases exhibit transglycosylation activity, by which β-1,3-oligosaccharides (glycosyl donors) are transferred to hydroxyl compounds (glycosyl acceptors), generating new glycosidic bonds [7]. The glycosyl acceptors include monosaccharides, oligosaccharides, and other hydroxyl-containing compounds, such as alcohols and amino acids [1]. New bonds can be synthesized during transglycosylation, which is important for the synthesis of new glycosides [8]. Numerous oligosaccharides, glycolipids, and glycoproteins with novel biological activities have been discovered through glycosyltransferase synthesis [9]. Therefore, research on glycosidases has attracted increasing attention in recent years.

Endo-1,3-β-glucanases are widely distributed in archaea [10], bacteria [11,12,13], viruses [14], and eukaryota [15,16,17,18]. According to the amino acid sequences, endo-1,3-β-glucanases are classified into seven glycoside hydrolase (GH) families: GH16, GH17, GH55, GH64, GH81, GH128, and GH152. These endo-1,3-β-glucanases function in various life processes, such as the degradation of polysaccharides for energy utilization, cell development, and cell differentiation. For example, the β-1,3-glucanase-related protein from the red swamp crayfish, *Procambarus clarkia,* can be induced by bacteria, which is an important factor in the immune response [19]. The killer toxin secreted by the yeast *Wickerhamomyces anomalus* has also exhibited β-glucanase activity, which has potential for use in the symbiotic control of malaria [20]. Marine organisms are important sources for deriving new endo-1,3-β-glucanases [1], especially the digestive tracts of marine invertebrates, which are usually composed of various enzymes related to polysaccharide utilization. More interestingly, the endo-1,3-β-glucanases from marine organisms have demonstrated increased transglycosylation ability relative to those from terrestrial sources [1]. To date, a total of ten endo-1,3-β-glucanases have been determined to exhibit transglycosylation activity, two of which belong to the GH17 family [18,21], and the other eight belonging to the GH16 family. Among these eight endo-1,3-β-glucanases, one is derived from marine bacteria [22], and the remaining are all derived from marine mollusks [16,17,23,24,25,26,27]. Due to the presence of both hydrolytic activity and transglycosylation activity, marine mollusk endo-1,3-β-glucanases have distinctive advantages in the preparation of novel, useful glucooligosaccharides or glucans. For instance, soluble β-1,3-1,6-glucan obtained by transglycosylation of laminarin, using the endo-1,3-β-glucanase derived from the scallop *Chlamys albidus,* exhibits high immune stimulation, and bacteriostatic and antitumor activities [16]. The reported marine mollusk endo-1,3-β-glucanases have shown another interesting feature: these enzymes exhibit diverse specificities, and even their amino acid sequences have a high degree of similarity [1]. To reveal their unique specificities, more research on marine mollusk endo-1,3-β-glucanases is required.

Previously, in combination with proteomic analysis and the use of the whole-genome database, we identified a novel endo-1,3-β-glucanase from a hepatopancreatic extract of the Zhikong scallop (*Chlamys farreri*) [28]. In this study, the gene of the endo-1,3-β-glucanase (named L_cf_) was cloned and expressed using the *Brevibacillus* (*Bacillus brevis*) expression system. After protein purification, the catalytic properties of L_cf_ were characterized, and the transglycosylation specificity was determined. This study enhanced our understanding of the marine mollusk endo-1,3-β-glucanases.

## 2. Results and Discussion

### 2.1. Sequence Alignment between L_cf_ and Endo-1,3-β-Glucanases from GH16

**Figure 1:**
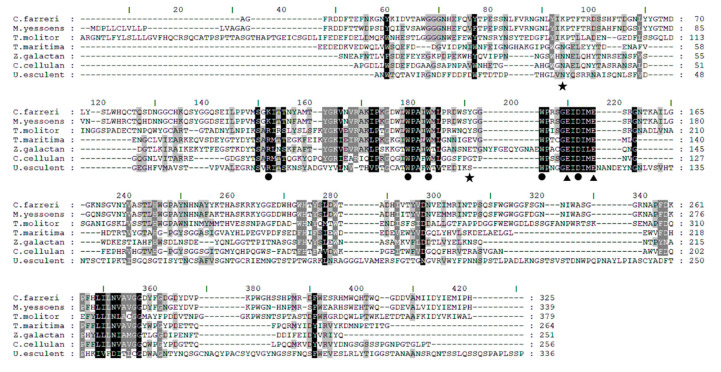
Sequence alignment of characterized GH16 β-1,3-glucanases from different organisms. The identical residues are highlighted in black, and similar residues are shaded in gray. The two key catalytic residues conserved in GH16 endo-1,3-β-glucanases are marked with triangles. For β-glucanase ZgLamA_GH16_, the residues involved in substrate binding are marked with closed circles and stars. The residues marked with closed circles are conserved in these endo-1,3-β-glucanases, but the two residues marked with stars are not conserved. The GeneBank accession numbers of the present endo-1,3-β-glucanases are: *Chlamys farreri*: AMN92714.1 (L_cf_); *Mizuhopecten yessoensis*: AAW34372.1; *Tenebrio molitor*: ACS36221.1; *Thermotoga maritima*: AAD35118.1; *Zobellia galactanivorans*: CAZ96583.1 (PDB ID: 4BOW); *Cellulosimicrobium cellulans*: AAC38290.1; and *Ustilago esculenta*: BAM29293.1.

Sequence alignment between L_cf_ and six characterized GH16 endo-1,3-β-glucanases was performed using ClustalW Multiple Alignment. These enzymes were derived from the marine mollusk *Mizuhopecten yessoensis* [29], terrestrial arthropod *Tenebrio molitor* [30], marine hot spring bacterium *Thermotoga maritima* [12], bacterium *Zobellia galactanivorans* [31] isolated from marine red algae, terrestrial bacterium *Cellulosimicrobium cellulans* [11], and terrestrial fungus *Ustilago esculenta* [32]. Sequence alignment indicated that L_cf_ shared 87.4% sequence identity with the endo-1,3-β-glucanases derived from the marine mollusk *Mizuhopecten yessoensis*. In contrast, L_cf_ shared only 23.6–41.3% sequence identity with the other endo-1,3-β-glucanases (Figure 1). This was consistent with the conclusion that endo-1,3-β-glucanases derived from marine mollusks have high homology [15,24].

Additionally, sequence alignment demonstrated the key residues involved in catalytic reactions and substrate binding. A previous study had demonstrated that two conserved glutamates in the pattern EXDX(X)E function in the catalytic reaction were a nucleophile and general acid/base, respectively [31]. In L_cf_, the equivalent to the nucleophile was Glu150, whereas the general acid/base was Glu155 (Figure 1). In addition, the structures of the β-glucanase ZgLamA_GH16_ complex with oligo substrates has previously been solved (PDB ID: 4BOW) [31], providing knowledge of key residues involved in substrate binding. The complex structures of ZgLamA_GH16_ identified seven residues involved in substrate binding, five of which are conserved based on the sequence alignment. In L_cf_, the equivalent to the conserved residues were Lys105, Trp130, Trp134, Asp152, and Trp145 (Figure 1).

### 2.2. Expression and Purification of L_cf_

**Figure 2:**
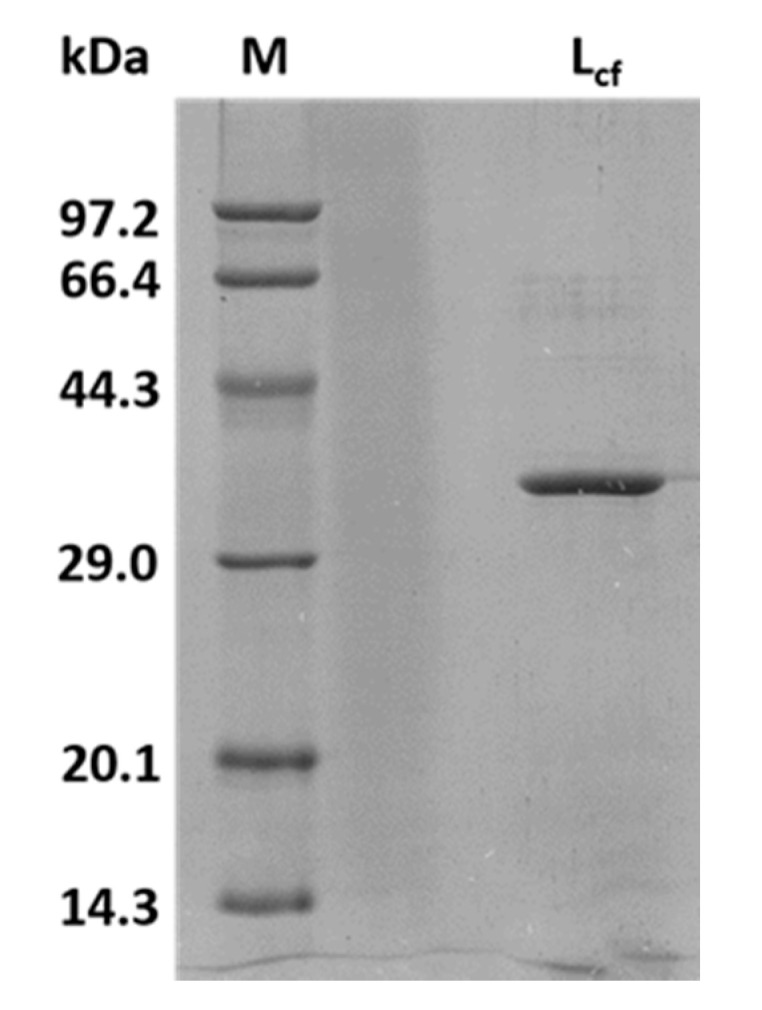
SDS-PAGE analysis of the purified L_cf_.

In a previous study, direct extraction of the enzyme from the digestive organs of marine mollusks was the general method used for collecting endo-1,3-β-glucanases [24,25,26]. However, the isolation of endo-1,3-β-glucanases from digestive organs is not only time-consuming, but is also limited by the source of marine mollusks. Therefore, we carried out the expression of L_cf_ in vitro using the *Escherichia coli* expression system and *Bacillus brevis* expression system. Although the recombinant proteins were detected in the supernatant of the cell lysis, they did not exhibit enzymatic activity, suggesting the occurrence of incorrect protein folding in the expression of L_cf_ in the *E. coli* expression system. Fortunately, recombinant L_cf_ with hydrolytic activity was detected successfully in the fermentation broth of *Bacillus brevis*. After a three-step purification, including ammonium sulfate salting out, hydrophobic chromatography, and ion exchange chromatography, about 12 mg of L_cf_ was extracted from 1 L of fermentation broth of *Bacillus brevis*. The molecular weight of L_cf_ was about 37 kDa, as shown in Figure 2.

Through the *Bacillus brevis* expression system, L_cf_ was expressed in vitro successfully, providing a more convenient method for the extraction of marine mollusk endo-1,3-β-glucanases. In addition, this method may facilitate performing further structural research, which will be important for understanding the unique transglycosylation activity of marine mollusk endo-1,3-β-glucanases.

### 2.3. Enzymatic Characterization of L_cf_

**Figure 3:**
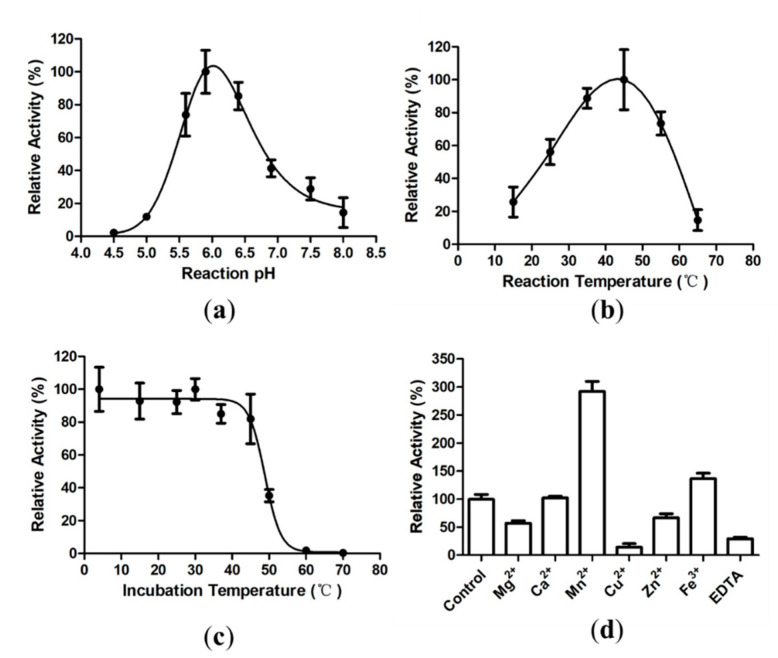
Effect of pH, temperature, and metal ions on L_cf_ enzymatic activity and the thermostability of L_cf_. (**a**) The effect of pH on L_cf_ activity. The optimal pH was determined by measuring the enzymatic activity of L_cf_ at pH 4.5, 5.0, 5.6, 5.9, 6.4, 6.9, 7.5, and 8.0, at 37 °C. (**b**) The effect of temperature on L_cf_ activity. The optimal temperature was determined by measuring the enzymatic activity of L_cf_ at 15 °C, 25 °C, 35 °C, 45 °C, 55 °C, and 65 °C. (**c**) The thermostability of L_cf_. The activity of L_cf_ was measured at 37 °C after pre-incubation at 4–70 °C for 15 min. (**d**) The effect of metal ions on L_cf_ activity. The activity of L_cf_ was measured in the presence of various metal ions (5 mM).

A hydrolytic activity assay at various pH values indicated that L_cf_ exhibited the maximum hydrolytic activity at pH 6.0 (Figure 3a). Additionally, the assay at different temperatures demonstrated that the optimal temperature for L_cf_ was 44 °C (Figure 3b). When the incubation temperature was higher than 45 °C, the hydrolytic activity of L_cf_ decreased significantly (Figure 3c). The enzymatic activity of L_cf_ was abolished after pre-incubation of L_cf_ at 60 °C and 70 °C (Figure 3c). In addition, L_cf_ retained approximately 25% of the enzymatic activity with the addition of EDTA (Figure 3d), suggesting that metal ions might influence the enzymatic activity of L_cf_, but are not essential for L_cf_. Notably, the enzymatic activity of L_cf_ was increased by approximately three times in the presence of Mn^2+^. The enzymatic activity of L_cf_ was also enhanced by Fe^3+^, and the addition of Ca^2+^ seemed not to affect the enzymatic activity of L_cf_. However, the enzymatic activity of L_cf_ was inhibited in the presence of Mg^2+^, Cu^2+^, and Zn^2+^, especially in the presence of Cu^2+^ (Figure 3d).

Furthermore, the kinetic parameters of L_cf_ towards laminarin were measured. The specific activity for L_cf_ was 1.67 U/mg, and its *K_m_* and *K_cat_* values were 10.27 mg/mL and 1230.64 S^−1^, respectively.

### 2.4. Analyses of Hydrolytic Products and Transglycosylation Products

**Figure 4:**
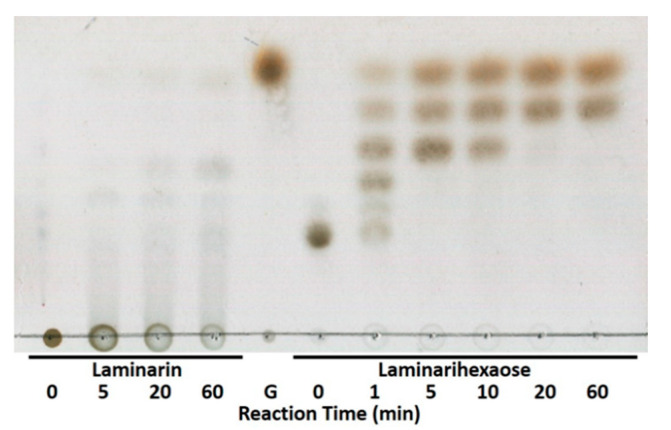
Thin layer chromatography (TLC) analysis of the hydrolysis products of laminarin and laminarihexaose using glucose (G) as control.

To test the hydrolytic products of L_cf_, Thin layer chromatography (TLC) analysis was carried out. As shown in Figure 4, both the laminarin and the laminarinexaose were hydrolyzed into several oligomers, confirming that L_cf_ acted in an endo-type mode. However, different cleavage efficiencies were observed. L_cf_ showed a more efficient cleavage rate toward laminarinexaose. After reaction for 1 min, hydrolysis products with a degree of polymerization (Dp) 1–5 were generated. These oligomeric products were further hydrolyzed into monomers and dimers as end-products (Figure 4). In contrast, laminarin was not hydrolyzed completely (Figure 4). Considering the fact that laminarins from *Laminaria digitate* have a branch degree of 7.68%, and these branches contain about 9.51% β-1,6-glycosidic bonds [33], the more complex structure and the longer chain of laminarins might lead to a lower cleavage rate for L_cf_.

**Figure 5:**
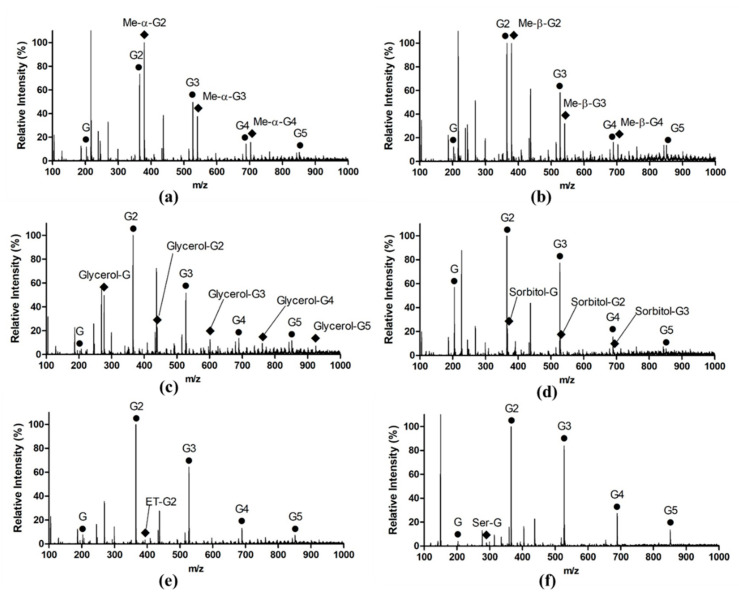
ESI-MS spectrometry analysis of the transglycosylation products. Laminarihexaose was used as the substrate or the donor. A total of six acceptors were tested in the transglycosylation reaction, including methyl α-d-glucopyranoside (**a**), methyl β-d-glucopyranoside (**b**), glycerol (**c**), d-sorbitol (**d**), ethanol (**e**), and l-Serine (**f**). The m/z of the ion peaks [M + Na]^+^ of the products are indicated by dots (hydrolysis products) and rhombuses (transglycosylation products). The m/z of the products are summarized in Table 1.

To explore the transglycosylation specificity of L_cf_, the transglycosylation products were analyzed by MS. Laminarin has usually been selected as the substrate for transglycosylation reactions in previous studies [16,23,24]. However, L_cf_ showed a more efficient cleavage rate toward laminarinexaose than laminarin, as described above. Accordingly, the substrate we used in the transglycosylation reaction was laminarinexaose. Furthermore, the acceptors contained monosaccharides (methyl α-d-glucopyranoside and methyl β-d-glucopyranoside), alcohol (ethanol), polyols (glycerol and d-sorbitol), and amino acids with hydroxyl side chains (l-serine). As shown in Figure 5a, the methylated disaccharide, trisaccharide, and tetrasaccharide were detected in the presence of methyl α-d-glucopyranoside or methyl β-d-glucopyranoside, suggesting that the donors of glycosylation were glucose, laminaribiose, and laminaritriose. Transglycosylation products were also observed in the presence of the other acceptors (Figure 5c–f). In the presence of glycerol, five transglycosylation products were generated (Figure 5c). Similar to methyl α-d-glucopyranoside or methyl β-d-glucopyranoside, three transglycosylation products were observed in the presence of d-sorbitol (Figure 5d). However, only one transglycosylation product was detected using ethanol or l-serine as the acceptor (Figure 5e,f). Besides transglycosylation products, hydrolytic products, including glucose, G2, G3, G4, and G5, were detected. The m/z of each product in ESI-MS (+) is shown in Table 1.

The transglycosylation activity of O-glycoside hydrolases can be used for the synthesis of new glycosides, which has potential for the development of novel drugs and functional foods. For example, transglycosylation may function in the synthesis of glycosylated therapeutic antibodies and glycoside-specific antibody–drug conjugates [34]. Therefore, the diverse transglycosylation specificities of endo-1,3-β-glucanases have attracted much attention. As described above, L_cf_ exhibited transglycosylation activity towards different kinds of acceptors, which facilitated the synthesis of novel glycosides. Additionally, in the transglycosylation reaction catalyzed by the endo-1,3-β-glucanase from *Mizuhopecten yessoensis*, only a sorbitol-Glc_3_ product was detected when using d-sorbitol as an acceptor [24]. However, three transglycosylation products linked with sorbitol were observed after the transglycosylation reaction catalyzed by L_cf_ (Figure 5d). These findings demonstrated that the two endo-1,3-β-glucanases from mollusks exhibit different transglycosylation specificities, although they share a high sequence identity. The unique transglycosylation activity of L_cf_ towards sorbitol facilitates the development of glycoconjugates with intestinal functions, and noncariogenic sugars [35]. In sum, this study provided insights into novel endo-1,3-β-glucanases from marine mollusks, enhancing our understanding of the diverse enzymatic specificities of marine mollusk endo-1,3-β-glucanases.

## 3. Materials and Methods

### 3.1. RNA Extraction and Gene Cloning

The scallops were purchased from the Nanshan seafood market in Qingdao. The hepatopancreas was dissected by hand from the scallop viscera. Then, the hepatopancreas was ground into powder in liquid nitrogen. Next, the total RNA was extracted from the powder using the Mollusc RNA Kit (Omega Bio-tek, Norcross, GA, USA). A cDNA library of scallop hepatopancreas was constructed by reverse transcription, using the extracted total RNA as a template (RevertAid First Strand cDNA Synthesis Kit, Thermo Fisher Scientific, Waltham, MA, USA). Using the cDNA library as a template, a full-length gene of L_cf_ without the signal peptide was cloned using the following primers: a forward primer (5′-CGGGATCCGCAGGCTTCCGTGACGATTTCAC-3′) and a reverse primer (5′-CCGCTCGAGTCAATGAGGTATCATCTCTATGTAATC-3′). Target gene fragments were collected using the Gel Extraction Kit (Omega Bio-tek, Norcross, GA, USA), and ligated into the shuttle vector pNCMO2 (Takara, Dalian, China) using the restriction enzymes *BamH*I and *Xho*I. Then, the ligation solution was transformed into *E. coli* JM109. Expression plasmids were extracted using the Plasmid Mini Kit (Omega Bio-tek, Norcross, GA, USA).

### 3.2. Protein Expression in Brevibacillus

The *Brevibacillus* (*Bacillus brevis*) expression system (Takara, Dalian, China) is a prokaryotic expression system that is particularly effective in the production of secretory proteins [36]. The expression plasmids were transformed into *Brevibacillus*-competent cells using the new Tris-PEG (NTP) method, as described in the protocol. *Brevibacillus* transformant cells were grown in MTNm solid medium (glucose 10 g/L, polypeptone 10 g/L, beef powder 5 g/L, yeast extract 2 g/L, FeSO_4_·7H_2_O 10 mg/L, MnSO_4_·4H_2_O 10 mg/L, ZnSO_4_·7H_2_O 1 mg/L, MgCl_2_·6H_2_O 4.1 g/L, and neomycin 10 mg/L, pH 7.0) at 37 °C. DNA sequencing was performed to confirm the recombinant *Brevibacillus*. Then, the recombinant *Brevibacillus* cells were cultured in TMNm liquid medium (i.e., MTNm medium without MgCl_2_) at 32 °C for 48 h. SDS-PAGE analysis indicated that L_cf_ was successfully secreted into the fermentation broth.

### 3.3. Protein Purification

As described above, the *Brevibacillus* transformant cells were cultured for 48 h, then the fermentation broth was harvested by centrifuging at 6000× *g* rpm for 5 min. To precipitate the enzymes, ammonium sulfate was slowly added to the fermentation broth to 60% saturation (0 °C). After 10 h, the precipitate was collected by centrifugation (12,000× *g*, 4 °C), followed by washing using ammonium sulfate solution (60% saturation, 0 °C). The resulting protein was re-dissolved in a Tris-HCl buffer (20 mM, pH 7.5) containing 15% saturation (0 °C) ammonium sulfate. Then, the protein was loaded onto Phenyl Sepharose beads (GE Healthcare, Boston, MA, USA), which were washed and eluted using Tris-HCl buffer (20 mM, pH 7.5) containing 15–0% saturation (0 °C) ammonium sulfate. After purification by the Phenyl Sepharose beads, the resulting protein was dialyzed into a sodium acetate buffer (50 mM, pH 5.0). Next, the protein was loaded onto CM Sepharose beads (GE Healthcare), which were washed and eluted using sodium acetate buffer (50 mM, pH 5.0) containing 0–0.5 M NaCl.

### 3.4. Hydrolytic Activity Assay

The hydrolytic activity of L_cf_ was determined by the modified DNS method [37]. The reaction mixture (200 μL), containing 0.1% (*w/w*) laminarin from *Laminaria digitate* dissolved in sodium acetate buffer (50 mM, pH 5.6) and 20 μL purified L_cf_, was incubated for 20 min at 37 °C. Then, 150 μL DNS was added to terminate the reaction. The resulting mixture was boiled for 5 min at 100 °C and measured at 520 nm. One unit (1 U) of hydrolytic activity was defined as the amount of enzyme required to produce the reducing sugar equivalent to 1 μmol of glucose per minute under the above reaction conditions.

The optimal pH of L_cf_ was determined by measuring the hydrolytic activity at different pH conditions. The buffers (50 mM) used for this assay contained sodium acetate buffers (pH 4.5, 5.0 and 5.6), sodium phosphate buffers (pH 5.9, 6.4 and 6.9), and Tris-HCl buffer (pH 7.5 and 8.0). The effect of the temperature was measured at 15–65 °C in 50 mM sodium acetate buffer, at pH 5.6. The above enzymatic activity was tested under standard conditions. To determine the thermal stability of L_cf_, the residual hydrolytic activity of L_cf_ (dissolved in sodium acetate buffer (50 mM, pH 5.6)) after incubation at different temperatures (4–70 °C) for 15 min was measured. The effect of metal ions was determined by measuring the activity with different metal ions (Mg^2+^, Ca^2+^, Mn^2+^, Cu^2+^, Zn^2+^, Fe^3+^, and EDTA) at 5 mM in 50 mM sodium acetate buffer, at pH 5.6. All the above assays were performed in triplicate.

### 3.5. TLC Assay

The products of L_cf_ degradation of laminarin and laminarihexaose (Megazyme) were generated as described above. The reaction mixture (200 μL) contained 0.1% (*w/w*) laminarin or laminarihexose dissolved in sodium acetate buffer (50 mM, pH 5.6) and 20 μL purified L_cf_. At different times, 5 μL of reaction mixture were taken for TLC analysis. Samples were spotted on silica gel 60 F_254_ plates (Merck, Darmatadt, Germany), and separated in a solvent of 1-butanol/acetic acid/water (2:1:1, *v/v*). To visualize the products, the plate was sprayed with a reagent containing 2 g of diphenylamine, 2 mL of aniline, 1 mL of HCl, 10 mL of H_3_PO_3_, and 100 mL of acetone, and was heated at 100 °C for 10 min [38].

### 3.6. Transglycosylation Products Assay

To explore the transglycosalating ability of L_cf_, several acceptors, including methyl α-d-glucopyranoside, methyl β-d-glucopyranoside, glycerol, d-sorbitol, ethanol, and L-serine, were involved in the transglycosylation reactions. Briefly, L_cf_ (0.02 U) was added into the mixture containing laminarihexaose (2 mg/mL) and an acceptor (2 mg/mL) dissolved in sodium acetate buffer (25 mM, pH 5.6). Then, the resulting mixture (200 μL) was incubated at 37 °C for 5 min. The reaction was terminated by the addition of an equal volume of 2.5% (*v/v*) aqueous ammonia. After centrifugation, the supernatant was collected and detected using positive ion electrospray ionization mass spectrometry (ESI-MS) (Agilent 6460 Triple Quad, Santa Clara, CA, USA).

## Figures and Tables

**Table 1:** Summary of m/z of the ion peaks [M + Na]^+^ corresponding to hydrolysis and transglycosylation products.

Symbol	Compound	m/z
G	Glucose	203.2
G2	Laminaribiose	365.4
G3	Laminaritriose	527.3
G4	Laminaritetraose	689.3
G5	Laminaripentaose	851.2
Me-α-G2	Methyl α-d-Glc2	379.4
Me-α-G3	Methyl α-d-Glc3	541.4
Me-α-G4	Methyl α-d-Glc4	703.2
Me-β-G2	Methyl β-d-Glc2	379.4
Me-β-G3	Methyl β-d-Glc3	541.3
Me-β-G4	Methyl β-d-Glc4	703.2
Glycerol-G	Glycerol-Glc1	227.3
Glycerol-G2	Glycerol-Glc2	439.4
Glycerol-G3	Glycerol-Glc3	601.2
Glycerol-G4	Glycerol-Glc4	763.2
Glycerol-G5	Glycerol-Glc5	924.9
Sorbitol-G	Sorbitol-Glc1	367.4
Sorbitol-G2	Sorbitol-Glc2	529.3
Sorbitol-G3	Sorbitol-Glc3	691.2
ET-G2	Ethanol-Glc2	393.3
Ser-G	Serine-Glc1	290.2
